# Identification of Chinese Herbs Using a Sequencing-Free Nanostructured Electrochemical DNA Biosensor

**DOI:** 10.3390/s151229773

**Published:** 2015-11-30

**Authors:** Yan Lei, Fan Yang, Lina Tang, Keli Chen, Guo-Jun Zhang

**Affiliations:** 1 School of Laboratory Medicine, Hubei University of Chinese Medicine, 1 Huangjia Lake West Road, Wuhan 430065, China; yanlei91316@163.com (Y.L.); Yangf1013@163.com (F.Y.); hjtanglina@163.com (L.T.); 2 School of Pharmacy, Hubei University of Chinese Medicine, 1 Huangjia Lake West Road, Wuhan 430065, China

**Keywords:** electrochemical biosensor, DNA identification, Chinese herb, *Fritillaria*, nanomaterials-modified electrode

## Abstract

Due to the nearly identical phenotypes and chemical constituents, it is often very challenging to accurately differentiate diverse species of a Chinese herbal genus. Although technologies including DNA barcoding have been introduced to help address this problem, they are generally time-consuming and require expensive sequencing. Herein, we present a simple sequencing-free electrochemical biosensor, which enables easy differentiation between two closely related *Fritillaria* species. To improve its differentiation capability using trace amounts of DNA sample available from herbal extracts, a stepwise electrochemical deposition of reduced graphene oxide (RGO) and gold nanoparticles (AuNPs) was adopted to engineer a synergistic nanostructured sensing interface. By using such a nanofeatured electrochemical DNA (E-DNA) biosensor, two Chinese herbal species of *Fritillaria* (*F. thunbergii* and *F. cirrhosa*) were successfully discriminated at the DNA level, because a fragment of 16-mer sequence at the spacer region of the 5S-rRNA only exists in *F. thunbergii*. This E-DNA sensor was capable of identifying the target sequence in the range from 100 fM to 10 nM, and a detection limit as low as 11.7 fM (S/N = 3) was obtained. Importantly, this sensor was applied to detect the unique fragment of the PCR products amplified from *F. thunbergii* and *F. cirrhosa*, respectively. We anticipate that such a direct, sequencing-free sensing mode will ultimately pave the way towards a new generation of herb-identification strategies.

## 1. Introduction

For a long time, Chinese herbal medicine has been an important component in the holistic therapy application of modern clinical medicine. Its effectiveness and safety rely pretty much on the proper choice of herbs, which is severely affected by the availability of substandard or non-medicinal herbs in the market. For instance, medicinal plant bulbs of the genus *Fritillaria* are used for their lung-moistening, cough-relieving and phlegm-dissolving effects. However, the efficacy varies even within the ten species of the *Fritillaria* genus recorded as original medicinal herbs in the Chinese Pharmacopoeia [1]. Unfortunately, they look identical or have highly similar chemical compositions, thus it is very hard to differentiate them. Although the emerging DNA barcode technology is capable of identifying the two *Fritillaria* species *F. thunbergii* and *F. cirrhosa* [2,3], it requires tedious sequencing of a large amount of samples, More importantly, its complicated data analysis needs a reference genome, which is not readily available in public databases. Therefore, the development of a simple, reliable and straightforward genotyping approach for identification of *F. thunbergii* and *F. cirrhosa* herbs is highly desirable to address these issues, if possible, at the specific DNA sequence level.

So far, sensors for DNA detection have been developed based on a variety of platforms, including field effect transistor [4,5], surface plasmon resonance spectroscopy [6], fluorescence [7,8], and electrochemical methods [9,10]. For example, electrochemical DNA (E-DNA) sensors have been widely used to perform genetic diagnostics [11], DNA hybridization [12], and determination of electrochemical active molecules in Chinese herbal medicines [13,14]. We note that electrochemical sensing technology has special advantages over other biosensors, such as low cost, simple operation, high sensitivity and specificity [15], which inspired us to construct a simple and sensitive E-DNA sensor to quickly probe the special DNA sequences for identification of *F. thunbergii* and *F. cirrhosa*. Generally, to improve sensitivity of an electrochemical sensor, graphene and gold nanoparticles (AuNPs) are often employed to construct E-DNA sensors owing primarily to their excellent conductivity, large surface area, remarkable electrocatalysis capacity and exceptional mechanical properties [16,17,18,19]. They can work cooperatively to enlarge the current response, resulting in increased sensitivity [20,21]. However, such a superior nanostructured signal amplification platform has not been applied in the identification of subtypes of Chinese herbs.

In this work, we constructed an E-DNA sensor with bottom-up electrodeposited nanocomposites, aiming to generate a rapid, accurate and quantitative readout of the target DNA in a sequencing-free fashion for direct discrimination between two species of the *Fritillaria* genus, *F. thunbergii* and *F. cirrhosa*. The nanoassembled sensing interface is readily fabricated via controllable electrochemical deposition of graphene and AuNPs, which clearly enhances the electron-transfer efficiency of the electrochemical redox molecule methylene blue (MB) [22,23]. A layer of thiol-ended DNA probe is immobilized on the AuNPs surface via Au-S chemistry [24], which is perfectly complementary with the a 16-mer unique sequence in the spacer region of the 5S-rRNA of *F. thunbergii*. The hybridization event between the probe and the target sequences/single-stranded DNA amplified by asymmetric polymerase chain reaction (PCR) from real samples, allows direct readout of the two Chinese herbal *Fritillaria* species. Compared to the existing genosensors, the currently-developed E-DNA sensor has the following advantages: (1) the mature E-DNA sensing interface is readily fabricated via rapid, controllable and environmentally friendly electrochemical deposition rather than traditional toxic chemical reduction or time-consuming drop-casting method; (2) the low-cost, sequencing-free E-DNA sensor is capable of achieving a simple and straightforward readout of inherent discrimination between Chinese herbal *Fritillaria* species (*F. thunbergii* and *F. cirrhosa*); (3) the developed E-DNA sensor shows a potential of multiplexed species-identification by using an integrated electrode array biosensor, leading to a promising direction in the authentication of confusable Chinese herb species in the future.

## 2. Materials and Methods

### 2.1. Apparatus and Reagents

All electrochemical experiments were performed on a CHI 660D electrochemical work station (ChenHua Instruments, Shanghai, China) equipped with a traditional three-electrode system, which consisted of a bare or modified glassy carbon electrode (GCE, 3 mm in diameter) as the working electrode, a Ag/AgCl (1.0 M KCl) electrode as the reference electrode and a platinum wire as the auxiliary electrode. The morphological properties of the nano-materials were characterized on a SIGMA field emission scanning electron microscopic (FE-SEM, Zeiss, Oberkochen, Germany). The extracted total DNA and PCR products were analyzed by agarose gel electrophoresis on a JY1000C electrophoresis apparatus (Junyi Dongfang Electrophoresis Instrument Co. Ltd., Beijing, China), and visualized under a ZF1-II ultraviolet transmission analyzer (Jiapeng Technology Co. Ltd., Shanghai, China).

Graphite powder (99.95%, 325 mesh) was purchased from Alfa Aesar Co. Ltd. (Tianjin, China). HAuCl_4_·3H_2_O, 6-mercapto-1-hexanol (MCH) and tris(2-carboxyethyl)phosphine hydrochloride (TCEP) were purchased from Sigma-Aldrich (St. Louis, MO, USA). Methylene Blue (MB) was purchased from Sinopharm Chemical Reagent Co., Ltd (Shanghai, China). Formamide and agarose gel were bought from Jierui Biological Engineering Co., Ltd (Shanghai, China). All other chemicals used were analytical reagent-grade unless otherwise specified. Ultrapure water was generated by a Milli-Q System (Millipore, Billerica, MA, USA).

Plant genomic DNA kit (DP305) was purchased from Tiangen Biotech Co., Ltd. (Beijing, China). DNA oligonucleotides (purified by HPLC) were purchased from Shanghai Sangon Biological Engineering Technological Co. Ltd. (Shanghai, China). Their base sequences are listed as follows. Probe DNA: 5′-HS-(CH_2_)_6_-CACAAAACGGGGGCGG-3′, target DNA: 5′-CCGCCCCCGTTTTGTG-3′, one-base mismatch DNA: 5′-CCGCCCCCATTTTGTG-3′, non-complementary DNA: 5′-CACAAAA CGGGGGCGG-3′, forward primer: 5′-GGATTCGTGCTTGGGCGA GAGTAGTA-3′, reverse primer: 5′-ACGCTAGTATGGTCGTGATTCCTAGG-3′.

### 2.2. Fabrication of the Graphene and Gold Nanoparticles Modified Glassy Carbon Electrode

Prior to fabrication, the bare GCE was polished sequentially with 1.0, 0.3 and 0.05 μm alumina slurries, then sonicated in ultrapure water and ethanol, respectively. Graphene oxide (GO) was synthesized via the classical Hummer’s method [25,26]. Next the obtained GO was used to synthesize reduced graphene oxide (RGO) on the surface of the bare GCE through one-step electrochemical reduction method by using cyclic voltammetry (CV) [27]. The detailed procedures have been already reported in our group’s previous work [10,28]. The RGO/GCE was immersed in 3 mM HAuCl_4_ and 0.1 M KNO_3_ solution as a working electrode and chronoamperometry was employed to obtain AuNPs by electrochemical deposition for 50 s at −0.2 V according to the previous protocol [11,16] with a few modifications. Afterwards, the AuNPs/RGO/GCE was dried with nitrogen and stored at a 4 °C refrigerator until use.

### 2.3. Probe Immobilization and Hybridization

Before dropping, the capture probe was treated with TCEP for 1 h to avoid disulfide bond formation [29]. Subsequently, 5 μL of 5 μΜ probe DNA was dropped on the surface of the cleaned AuNPs/RGO/GCE and incubated at 4 °C overnight, through which probe DNA was covalently immobilized on the AuNPs/RGO/GCE surface via Au-S binding. After that, the DNA-modified electrode was washed by 1 × PBS and pure water in turn to remove the unbound probe. Hence, the probe DNA-modified electrode was named as ssDNA/AuNPs/RGO/GCE. Then the DNA-modified electrode was immersed into 2 mM MCH for 1 h to fill the unoccupied region. After rinsed thoroughly with deionized water and dried with nitrogen, the hybridization process was performed by incubating various concentrations of complementary DNA on the probe modified electrode surface and incubated for 40 min at 37 °C [23]. After hybridization, the modified electrode was denoted as dsDNA/AuNPs/RGO/GCE. Then the dsDNA-modified electrode was rinsed with ultrapure water and 1 × PBS containing 0.1% sodium dodecyl sulfate (SDS) twice to remove the unhybridized target DNA.

### 2.4. Accumulation of MB and Electrochemical Measurements

After hybridization, the above modified electrode was immersed into the stirring 20 μM MB dissolved in 25 mM NaCl solution for 5 min [10] to allow MB to accumulate onto the surface-hybridized dsDNA. Subsequently, the electrode was cleaned carefully with 0.1% SDS solution and water to prevent the physisorption of MB. After dried with N_2_ flow, the electrode was immersed into 20 mM Tris-HCl buffer (pH 7.0) as a working electrode for the electrochemical detection by differential pulse voltammetry (DPV). The reduction peak current caused by MB accumulation is closely correlated with the amount of target DNA hybridized with the probe. Therefore, the signal change (Δ*I* = *I*_dsDNA_ − *I*_ssDNA_) before (*I*_ssDNA_) and after (*I*_dsDNA_) hybridization could be used for quantification [16,23]. The DPV measurement parameters were as follows: potential range: 0.1~−0.6 V; amplitude: 0.05 V; pulse width: 0.05 s; sample width: 0.02 s; pulse period: 0.2 s and quiet time: 2 s.

### 2.5. Preparation of DNA Samples and Asymmetric Polymerase Chain Reaction (PCR) Amplification

Fresh leaves of *F. thunbergii* (which were respectively obtained from Pan’an Country (c1), Zhejiang Province; Linzhi Country (c2), Xizang Autonomous Region; Anguo Chinese herbal medicine market (c3), Hebei Province) and *F. cirrhosa* (Hehuachi Chinese herbal medicine market (b), Chengdu. Sichuan Province) were ground into powder in liquid nitrogen. DNA extraction was conducted using a plant genomic DNA kit following the manufacturer’s instructions. The purity of extracted genomic DNA was evaluated by measuring OD_260_/OD_280_ value using a spectrometer. Then the DNA stock solution was kept frozen before next PCR process.

The asymmetric PCR was performed as described before [30] with a minor change. The reaction system was in a final volume of 50 μL mixture, which contained 1 μL of formamide, 5 μL of 10 × PCR buffer (200 mM Tris-HCl, pH 8.4, 500 mM KCl), 4 μL of 25 mM MgCl_2_, 2 μL of DNA extracted as template, 1 μL of 10 mM dNTPs, 2.5 μL of 0.1 μM forward primer, 2.5 μL of 10 μM reverse primer, 1.5 μL of 1 units/μL Taq DNA polymerase (Shanghai Sangon Biotech Co. Ltd., Shanghai, China), and 30.5 μL sterilized double-deionized water. Finally, the mixed liquids were blocked with 25 μL paraffin oil. PCR was performed on a thermal cycler (Bio-RAD T100^TM^, Bio-Legend BioTech Co. Ltd., Shanghai, China) with the following settings: 4 min at 95 °C for initial denaturation, followed by 40 cycles of 95 °C for 30 s (denaturation), 60 °C for 30 s (annealing), 72 °C for 45 s (extension), Final extension was performed at 72 °C for 10 min, then the reaction was hold at 4 °C. To analyze, 7 μL of PCR products and 3 μL of SYBR Green I (Shanghai GENEray Biotech Co. Ltd., Shanghai, China) containing autoclaved water and 5 × loading buffer (1:99:150) were loaded in 1.5% agarose gel prepared with 1 × TAE buffer at 110 V for 1 h and observed under ultraviolet light.

## 3. Results and Discussion

### 3.1. Tailored Electrochemical Sensor for Sequence-Specific Detection of Fritillaria DNA

**Scheme 1:**
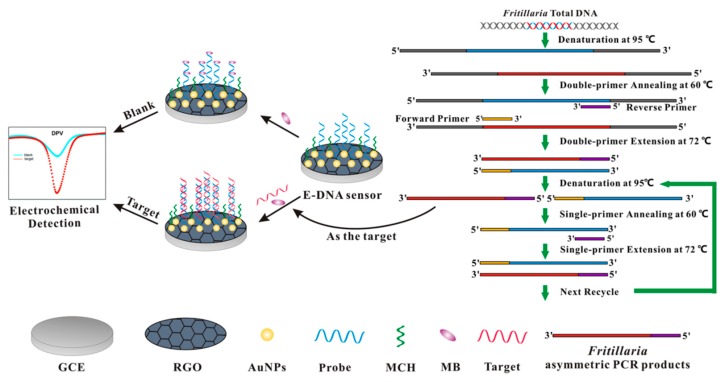
Schematic illustration of the fabrication process for the E-DNA biosensor and the asymmetric PCR procedure.

A nanostructured E-DNA biosensor based on graphene-AuNPs functionalization for the sequence-specific detection of DNA sequences in herbal plant (*Fritillaria* species) is illustrated in Scheme 1. RGO is first assembled on the surface of GCE by one-step electrochemical reduction method to enhance the electrochemical response. Then AuNPs are electrodeposited on the RGO layer for further signal amplification [31,32]. Prior to detection, capture probe DNA is tethered onto the AuNPs surface via a strong Au-S bond, followed by MCH blocking to fill the unoccupied region, which renders the probe a favorable orientation. To facilitate the next rapid and accurate sequence-specific readout of target DNA, and even PCR amplicons in complex matrices, several issues need to be addressed. Firstly, to reduce the assay cost and simplify the procedure, the redox MB is employed as a straightforward, label-free and cost-effective electrochemical indicator to generate the readable DPV signals, showing remarkable current changes before and after DNA hybridization. Secondly, the core parameters of this E-DNA sensor, including sensitivity, selectivity, reproducibility and stability, require an optimization by using the designed standard target DNA. Thirdly, to distinguish the *F. thunbergii* and *F. cirrhosa*, asymmetric PCR based on different amounts of double-primer is used to yield single-stranded DNA (ssDNA) as targets. The detailed procedure involves the following steps: (i) the total DNA of *Fritillaria* species plants is separately extracted for subsequent denaturation to single strands; (ii) after annealing, different amounts of double-primer are incorporated to produce double-stranded DNA (dsDNA); (iii) as the forward primer is exhausted, one strand of the amplified dsDNA can act as a template to amplify the desired target DNA with the assistance of reverse primer and manifold cycles. The yielded ssDNA target sequence could selectively hybridize with the probe and lead to electrochemical signal change for direct readout of identification information without complex (high throughput) sequencing.

### 3.2. Engineering Synergisitc Biosensing Interface

**Figure 1:**
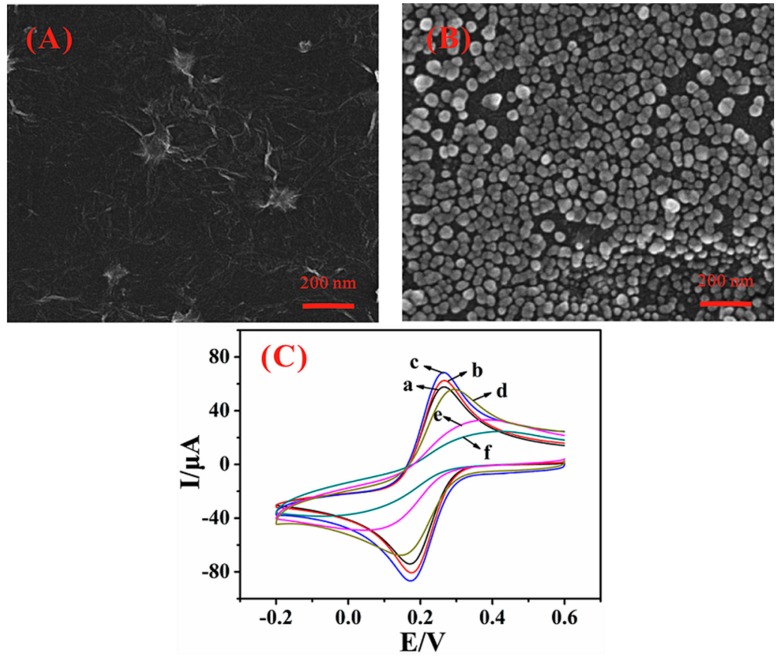
SEM images of (**A**) RGO/GCE and (**B**) AuNPs/RGO/GCE; (**C**) CV diagrams of 5 mM K_3_[Fe(CN)_6_] and 0.1 M KCl solution at different modified electrodes. Curves: (a) bare GCE; (b) RGO/GCE; (c) AuNPs/RGO/GCE; (d) ssDNA/AuNPs/RGO/GCE; (e) MCH/ssDNA/AuNPs/RGO/GCE; (f) dsDNA/AuNPs/RGO/GCE.

The morphologies of the RGO and the AuNPs/RGO nanocomposite were observed by SEM. As shown in Figure 1A, a typically wrinkled and crumpled sheet structure was obtained for the RGO, which is in accordance with the previously described report [33]. After electrodeposition, AuNPs were densely dispersed on the graphene surface with a diameter of approximately 30 nm (Figure 1B), confirming that AuNPs have been successfully fabricated. Furthermore, CV was employed to characterize the stepwise fabrication process of the electrochemical DNA biosensor, in which Fe(CN)_6_^3−/4−^ was chosen as a redox probe. As shown in Figure 1C, the peak current of bare GCE (curve a) was obviously smaller than that of RGO/GCE (curve b). After AuNPs were assembled on the surface of RGO/GCE, the peak current distinctly increased (curve c). All these enhancement consequences came from the excellent conductivity of graphene and AuNPs as reported [34,35]. When the probe DNA was modified on AuNPs/RGO/GCE via Au-S bonding, the peak current sharply decreased (curve d), and further declined after blocked by MCH (curve e). When the probe DNA was hybridized with target DNA, the conductivity of the electrode was clearly reduced (curve f), indicating that negatively charged phosphate backbone of DNA has limited the electron transfer of Fe(CN)_6_^3−/4−^ because of electrostatic repulsion interaction as dsDNA is more negatively charged than ssDNA [36].

### 3.3. Optimization of Experimental Conditions

**Figure 2:**
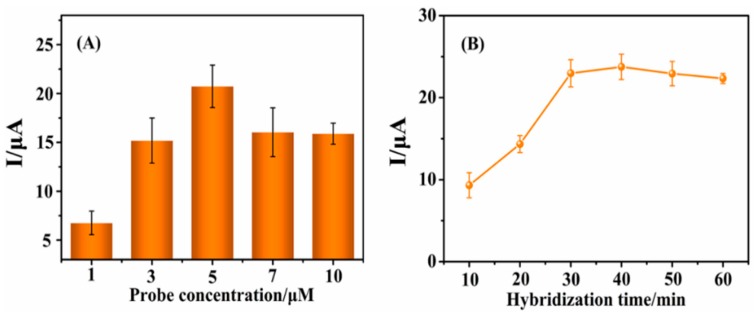
(**A**) Effect of the probe DNA concentration on DPV responses of the peak current of MB; (**B**) Effect of DNA hybridization time on DPV responses of the peak current of MB. (*C*_MB_ = 20 mM, *T*_hybridization_ = 37 °C, *C*_complementary DNA_ = 100 pM).

It is reported that experimental parameters such as probe concentration and hybridization time have great influence on the accuracy and sensitivity of an electrochemical sensor [37,38]. Hence, the influence of these two factors was investigated in this work. Figure 2A shows that the DPV peak current of MB increased with the probe concentrations ranging from 1 μM to 5 μΜ, then sharply decreased and tended to be saturated at 7 μΜ and 10 μΜ. Figure 2B shows the effect of hybridization time on the DPV response. With the increasing hybridization time from 10 min to 60 min, the reduction peak current suddenly enlarged and reached to a maximum value at 40 min, and then slightly decreased. As a result, 5 μΜ probe concentration and 40 min hybridization time were selected as the optimal conditions throughout the total experiment.

### 3.4. Sensitivity

**Figure 3:**
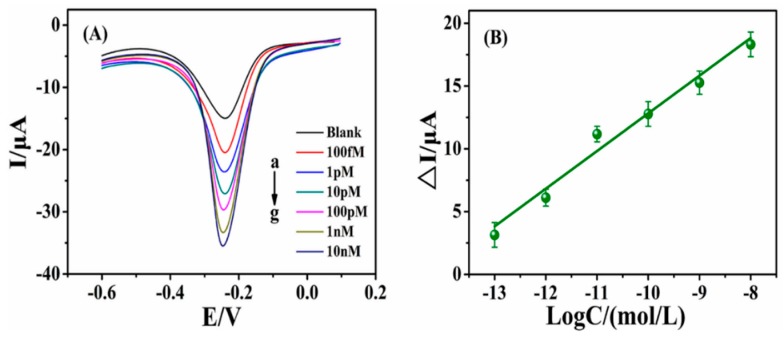
(**A**) DPV curves corresponding to the different detection concentrations of target DNA: 0, 100 fM, 1 pM, 10 pM, 100 pM, 1 nM, 10 nM, respectively; (**B**) Linear relationship of the reduction peak current with the target DNA concentration.

Under optimal experiment conditions, the sensitivity of the DNA sensor was explored by DPV. As seen in Figure 3A, the peak current of MB increased gradually with the concentrations of complementary target DNA concentrations ranging from 100 fM to 10 nM, and the linear correlation relationship between Δ*I* (*I*_dsDNA_ − *I*_ssDNA_) and the logarithm of target DNA concentration were displayed in Figure 3B. The linear regression equation was Δ*I* (μA) = 3.042 logC (M) + 42.90 (*R* = 0.9921). The detection limit was estimated to be 11.7 fM (S/N = 3). The comparison of this proposed method with other works is illustrated in Table S1. These results state that the designed electrochemical DNA sensor is more sensitive than most other sensors. This is probably because of the synergetic signal amplification caused by modification of AuNPs and RGO composite on the electrode. Du *et al.* fabricated an electrode with individual RGO material for DNA detection based on peptide nucleic acid-DNA hybridization, and achieved a lower sensitivity than that of this work. Subsequently, Zhang and Jiang developed the other DNA biosensor based on AuNPs/RGO. The sensitivity was improved by about one order of magnitude, but was still lower than that of this work. This may be ascribed to the short electro-deposition time (20 s) of AuNPs, leading to a low density of AuNPs for the probe immobilization. From the abovediscussed results, this nanostructured E-DNA biosensor has shown good sensitivity that could recognize low concentrations of target DNA easily.

### 3.5. Selectivity

**Figure 4:**
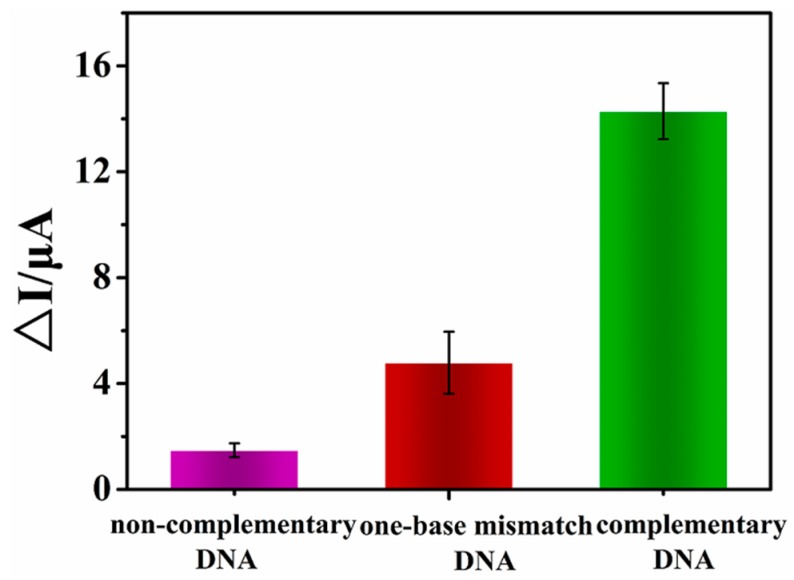
DPV peak current of 100 pM noncomplementary DNA; single-base mismatch DNA; complementary DNA.

As specificity is a very important property of a DNA biosensor, three different sequences including complementary DNA, single-base mismatch DNA, and non-complementary DNA, respectively, were employed to test this sensor under the same conditions. As shown in Figure 4, the Δ*I* (*I*_dsDNA_ − *I*_ssDNA_) value of complementary DNA was apparently higher than that of single-base mismatch DNA. When hybridized with non-complementary DNA, the response was found to be negligible. These results declare that the proposed electrochemical biosensor could distinguish single nucleotide from complementary target with excellent selectivity.

### 3.6. Reproducibility and Stability

In practical applications the reproducibility of a biosensor is an extremely vital standard to judge whether a biosensor is reliable or not. Herein, five parallel AuNPs/RGO composite modified electrodes were modified with probe DNA and applied to detect 10 pM target DNA, respectively, and the relative standard deviation (RSD) of the peak current was found to be 5.85%, suggesting that the biosensor has excellent reproducibility. The stability of the fabricated electrodes was also investigated by applying the DNA-modified electrodes with 10 pM complementary sequence and measuring the response of the sensor every day in 6 days. As shown in Figure S1, the DPV response changed negligibly within 6 days, denoting an excellent stability of this sensor.

### 3.7. Direct Identification of F. thunbergii and F. cirrhosa Using E-DNA Sensor

**Figure 5:**
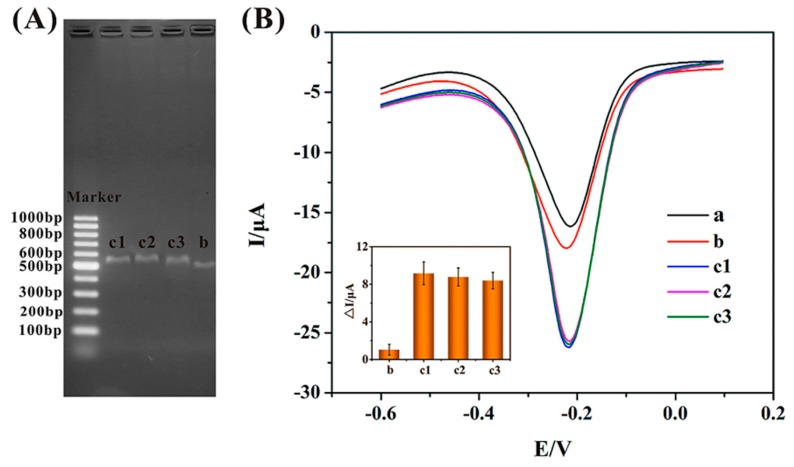
(**A**) Agarose gel electrophoresis diagram of asymmetric PCR products. From left to right: DL1002 marker, *F. thunbergii* (c1, c2 and c3), *F. cirrhosa* (b), respectively; (**B**) Differential pulse voltammograms of different PCR products exposed to Tris-HCl buffer blank control (a), and hybridized with *F. cirrhosa* (b), *F. thunbergii* (c1, c2 and c3). Inset: Histogram of pure peak current corresponding to *F. cirrhosa* (a) and different *F. thunbergii* (c1, c2 and c3).

To demonstrate whether the proposed electrochemical DNA sensor could be applied to differentiate real traditional Chinese medicinal plant samples selectively and accurately, asymmetric PCR products amplified from *F. thunbergii* and *F. cirrhosa* were used as detection targets. The agarose gel electrophoresis analysis diagram of the obtained PCR products was shown in Figure 5A. The PCR amplification products from *F. thunbergii* (band c1, c2 and c3) showed light bands between 500–600 bp, which correlates with the result (564 bp) reported in the literature [3]. However, the PCR products of *F. cirrhosa* (band b) appeared a band apparently at 500 bp, which is obviously different from that of *F. thunbergii*. This phenomenon might result from the deficiency of specific target DNA in *F. cirrhosa* genes to the immobilized probe. All these results are in good agreement with the previous report [30].

Before electrochemical detection, PCR products were heated in hot water (95 °C) for 5 min, and immediately chilled in ice for another 5 min to obtain denatured ssDNA. After that, the denatured PCR products were applied to the probe-modified electrodes for the electrochemical detection. Figure 5B reveals the DPV responses of the DNA biosensor to the different PCR amplified products. Curve a (*I* = 13.25 μA) was the blank control, in which the probe-immobilized electrochemical biosensor was incubated with 20 mM Tris-HCl buffer (pH 7.0) without any hybridization. After hybridization with the different PCR products and incubated in MB, the peak currents of the PCR products obtained from *F. thunbergii* (curve c1, c2 and c3) and *F. cirrhosa* (curve b) were 22.42 μA, 21.65 μA, 22.03 μA and 14.30 μA, respectively. The DPV responses of 3 kinds of *F. thunbergii* obtained from different locations were comparably similar, while the DPV responses of *F. thunbergii* were distinctly higher than that of *F. cirrhosa*. As the probe DNA assembled on the proposed sensor is specific to *F. thunbergii*, only the PCR products from this species could hybridize with the probe and cause remarkable signals, while the sequences of the PCR products from *F. cirrhosa* are not complementary to the immobilized probe, leading to smaller signals. These results indicate that the electrochemical DNA sensor could specifically distinguish two close species of *Fritillaria* by detecting the PCR products amplified from *F. thunbergii* and *F. cirrhosa*, which is in good agreement with the results obtained from the gel electrophoresis. Therefore, this kind of electrochemical DNA sensor has shown a great potential as the analysis technique for plant identification.

## 4. Conclusions

A nanosensing interface-based E-DNA biosensor has been developed for identification of *Fritillaria* species at DNA level in a sequencing-free manner. Both graphene and AuNPs were assembled on a GCE electrode by an electrochemical reduction method, showing outstanding amplification properties. MB was utilized as a label-free indicator for the hybridization events. The proposed biosensor showed superior sensitivity (a detection limit of 11.7 fM), selectivity, reproducibility and stability, and was successfully applied to detect the asymmetric PCR products amplified from real *F. thunbergii* and *F. cirrhosa* samples, respectively. The results confirm that this sensor could distinguish these two close *Fritillaria* species accurately and reliably. Further, the obtained favorable linear range and low detection limit of target sequence can provide quantitative information, showing a great potential to carry out a direct, PCR-free identification of closely resembled subspecies in untreated herbal extracts. It is anticipated that such a differentiating strategy would be extended to achieve multiplexed Chinese herbal species-identification simultaneously by using a nanostructured electrode array biosensor as a complementation.

## Supplementary Files

Supplementary File 1
