# Supplementary material for: Identification of Chinese Herbs Using a Sequencing-Free Nanostructured Electrochemical DNA Biosensor
Source: Sensors (Basel). 2015 Nov 30;15(12):29882–92. doi: 10.3390/s151229773 (PMC4721694; doi:10.3390/s151229773)
Supplement: Supplementary File 1 [file sensors-15-29773-s001.pdf]

Supplementary Materials: Identification of Chinese Herbs Using a Sequencing-Free Nanostructured Electrochemical DNA Biosensor

Yan Lei [1,2], Fan Yang [1], Lina Tang [1], Keli Chen [2,*] and Guo-Jun Zhang [1,*]

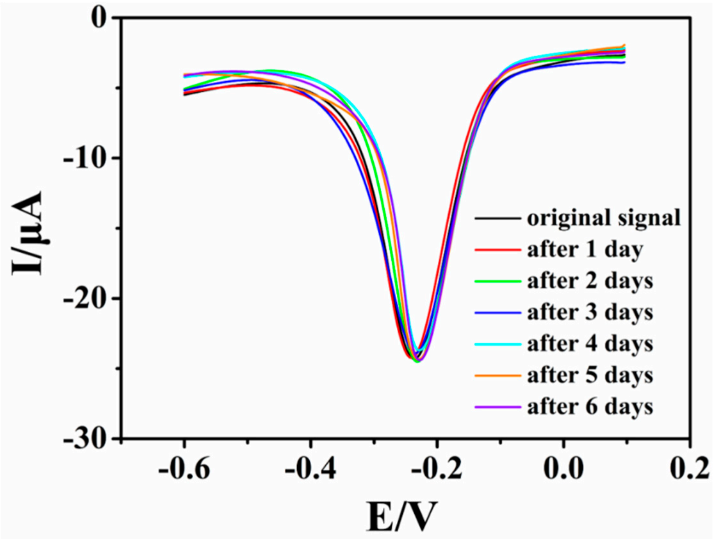

Figure S1. DPV diagrams of 10pM target DNA on AuNPs/RGO/GCE at different days.

Table S1. Comparison of the proposed method with other different modified electrodes for DNA detection.

Electrodes	Method	Linear Range	LOD (pM)	References
ERGO/GCE	DPV	1 pM–100 nM	0.545	[10]
AuNPs/rGO/GCE	DPV	0.1 pM–10 nM	0.035	[16]
AuNPs/PANI/CS-GS/GCE	DPV	10 pM–1 nM	2.11	[11]
AuNPs/pThion/graphene/GCE	DPV	0.1 pM–10 nM	2.11	[23]
AuNPs/RGO/GCE	DPV	100 fM–10 nM	0.0117	This work

Note: ERGO, electrochemical reduced graphene oxide; rGO, reduced graphene oxide; PANI, polyaniline; CS, chitosan; GS, graphene sheets; pThion, polythionine; GO, graphene oxide.
